# Phenotypic characteristics and plasmid structure differences between ST11 carbapenem-resistant *Klebsiella pneumoniae* and derived hypervirulent strains

**DOI:** 10.3389/fcimb.2026.1904182

**Published:** 2026-08-26

**Authors:** Na Du, Yongshi Zhao, Min Yang, Qiuyue He, Shumin Liu, Kai Yang, Min Niu, Yan Du

**Affiliations:** 1 Department of Clinical Laboratory, The First Affiliated Hospital of Kunming Medical University, Kunming, China; 2 Yunnan Key Laboratory of Laboratory Medicine, Kunming, China; 3 Yunnan Province Clinical Research Center for Laboratory Medicine, Kunming, China

**Keywords:** biofilm, blaKPC-2 gene, carbapenem, *Klebsiella pneumoniae*, pLVPK-like plasmid, resistance, virulence

## Abstract

Hypervirulent carbapenem-resistant *Klebsiella pneumoniae* (HCKP) strains cause infections of extremely high morbidity and mortality, and pose a great threat to public health. The number of reports of HCKP has increased in recent years. However, phenotypic characteristics, and structural features of the resistance and virulence plasmids carried by different geographic strains of HCKP may vary. We collected 18 carbapenem-resistant *Klebsiella pneumoniae* (CRKP) strains, including nine HCKP and nine non-HCKP (refers to carbapenem-resistant but non-hypervirulent *Klebsiella pneumoniae*) strains from a tertiary teaching hospital in Yunnan. Antimicrobial susceptibility tests showed that 18 strains exhibited high resistance to common clinical antibiotics. Diverse hypervirulence factors and resistance genes were identified, including *iuc*A, *iro*N, *iut*A, *ent*A, *ent*B, *fep*A, *fep*B, *iro*E, *irp*1, *irp*2, *ybt*Q, *ybt*S, *peg*-344, *rmp*A, *rmp*A2, *wca*J, *mrk*A, *mrk*B, *fim*A, *fim*B, *ure*A, *uge*, *wab*G, *bla*_SHV-12_, *bla*_TEM-1_, *bla*_KPC-2_, *sul*1, *tet*(A) and *tet*(R). All strains were of the ST11 type. KL64 and KL47 capsular serotypes were identified, with KL64 being the most prevalent type. The siderophore production ability of HCKP was higher than that of non-HCKP. Pulsed field gel electrophoresis (PFGE) separated five PFGE clusters among HCKP strains and seven among non-HCKP strains. Plasmid sequencing showed that the virulence plasmid featured a novel structure, with deletion of the *iro*BCD but retention of the *iro*N. The HCKP strain carried 4–6 plasmids, among which the structure of the pLVPK-like and *bla*_KPC-2_-bearing plasmid were relatively conserved, especially the sequences surrounding the hypervirulence and *bla*_KPC-2_ genes. Furthermore, the HCKP strains also harbored a drug-resistant conjugative plasmid with a highly conserved structure. The non-HCKP strains contained 2–4 plasmids, and the structure of the *bla*_KPC-2_-bearing plasmid showed high genetic diversity, with resistance factors flanked by the multiple insertion sequences, transposons and integrons. In the mouse intraperitoneal infection model, it was proved that the representative HCKP-67 strain showed stronger pathogenicity than CRKP-144 indicating that the distinction between HCKP and non-HCKP was correct. The implementation of effective prevention and control measures is urgently required to prevent further dissemination of such organisms.

## Introduction

*Klebsiella pneumoniae* (KP) is a nosocomial Gram-negative pathogen that causes severe infections, including pyogenic liver abscess, bacteremia, and urinary tract infection (Xu et al., 2024). *Klebsiella pneumoniae* strains can generally be classified into classical *Klebsiella pneumoniae* (cKP) and hypervirulent *Klebsiella pneumoniae* (hvKP). The cKP can cause invasive hospital-acquired infections among immunocompromised patients, and its virulence is relatively low, but resistance is easily acquired. hvKP often causes community-acquired infections in young and healthy hosts, and it exhibits a hypervirulence phenotype but is usually antibiotic-sensitive. Recently, a new type of “superbug”—hypervirulent carbapenem-resistant *Klebsiella pneumoniae* (HCKP)—has been widely reported worldwide; HCKP exhibits hypervirulence and carbapenem resistance and is associated with high morbidity and mortality (Yang et al., 2022). In July 2024, the World Health Organization warned of a global increase in HCKP infections (Sati et al., 2025). In recent years, HCKP, particularly the ST11 type, has been widely reported in clinical settings in China (Dong et al., 2022). Moreover, a report on a carbapenem-resistant *Klebsiella pneumoniae* (CRKP) surveillance in various countries showed that the prevalence of HCKP among CRKP strains in China is as high as 69% (Zhang et al., 2017). However, while some studies have already demonstrated the evolutionary path of HCKP, the factors that drive its high prevalence in clinical settings in China are unknown.

Mechanisms for the emergence of HCKP can be delineated through three evolutionary patterns (Tian et al., 2022): (i) a CRKP strain acquiring a virulence plasmid; (ii) a hvKP strain obtaining a resistance plasmid; and (iii) a KP strain acquiring a convergent plasmid carrying carbapenem resistance and hypervirulence genes. Karlson et al (Karlsson et al., 2019). reported a *bla*_KPC-2_-carrying sequence type (ST) 23 capsular serotype K1 hvKP isolate from the United States, and Chen et al (Chen et al., 2020). reported 18 *bla*_KPC-2_-carrying ST23, ST65, ST86, K1, K2, and K20 hvKP isolates from Singapore. Their research supports the first evolution pattern. Gu et al (Gu et al., 2018). reported five virulence plasmid-harboring ST11 CRKP strains from China, supporting the second evolution pattern. Moreover, Xie et al (Xie et al., 2021). identified fusion plasmid-carrying ST86 K2 HCKP isolates from China, which perfectly explains the third evolution pattern. The HCKP strain possesses different evolutionary mechanisms, leading to the carriage of plasmids with different structures. Furthermore, the prevalence of STs and capsular serotypes vary among countries and regions.

To date, very little research has been conducted on HCKP in Yun Nan, located in southwest China. In this study, we collected nine HCKP and nine non-HCKP strains from a tertiary teaching hospital in Yunnan through phenotypic testing combined with plasmid sequencing to compare the structural differences in plasmids between the two type strains. In doing so, we aimed to compare the phenotypic characteristics and plasmid structure differences between HCKP and non-HCKP. Our research will provide a theoretical basis for clinically curbing the spread of dual-threat “superbugs”.

## Materials and methods

### Bacterial isolates and identification

A total of 18 CRKP non-duplicated isolates were collected from a tertiary teaching hospital in Yunnan, China. CRKP was defined as a clinical strain with resistance to carbapenems (including imipenem, meropenem and ertapenem) according to the guidelines of the Clinical and Laboratory Standards Institute (CLSI) guidelines (CLSI, 2025). We confirmed the species identity of all isolates via matrix-assisted laser desorption/ionization mass spectrometry (MALDI-TOF/MS; BioMérieux, France). hvKP was defined as possessing some combination of the regulator of mucoid phenotype A (*rmp*A) and/or regulator of mucoid phenotype A2 (*rmp*A2), with the siderophore aerobactin (*iuc*A), salmochelin (*iro*B), or putative metabolite transporter (*peg*-344) (Li et al., 2023). The quality control strains used were *Enterobacter aerogenes* ATCC13048. All bacteria were stored at -80°Cin Luria-Bertani broth containing 20% glycerol.

### Antimicrobial susceptibility testing

Antibiotic susceptibility tests were performed for the isolates using the VITEK 2 Compact system (BioMérieux, France) or the disk diffusion method. The results were interpreted as recommended by the CLSI (CLSI, 2025). *Escherichia coli* ATCC 25922 served as the control in antimicrobial susceptibility testing.

### Detection of virulence and resistance genes

Genomic DNA was extracted from the CRKP strains using the boiling method. The primers used are listed in **Supplementary Table 1**. Virulence genes included iron uptake system-related genes (*iuc*A, *iroN*, *iut*A, *ent*A, *ent*B, *fep*A, *fep*B, *iro*E, *irp*1, *irp*2, *ybt*Q, *ybt*S), the putative metabolite transporter (*peg*-344), adhesion regulator (*rmp*A, *rmp*A2), capsule synthesis gene (*wca*J), type III fimbrial-related genes (*mrk*A, *mrk*B), type I fimbrial-related genes (*fim*A, *fim*B), urease-related genes (*ure*A) and lipopolysaccharide-related genes (*uge*, *wab*G). Among them, the *peg*-344, *iuc*A, *rmp*A, and *rmp*A2 are characteristic genes of the hypervirulent phenotype. Resistance genes included extended-spectrum β-lactamase genes (*bla*_SHV-12_, *bla*_TEM-1_), class A carbapenemase gene (*bla*_KPC-2_), class B carbapenemase genes (*bla*_NDM-1_), class D carbapenemase genes (*bla*_OXA-48_), the sulfonamide resistance gene (*sul*1), and tetracycline efflux pump genes (*tet(*A), *tet(*R)). All the above-mentioned genes were detected by polymerase chain reaction (PCR) using specific primers. The amplification conditions were as follows: pre-denaturation at 94°C for 10min, denaturation at 94°C for 30 s, annealing at 58°C for 30 s, extension at 72°C for 30 s, 35 cycles, extension at 72°C for 10min, and hold at 4°C. Subsequently, PCR products were visualized by agarose (2%) gel electrophoresis, and the representative amplified positive PCR products were further confirmed by direct DNA sequencing (Sangon Biotech, Shanghai, China). Nucleotide sequences were compared using the Basic Local Alignment Search Tool (http://blast.ncbi.nlm.nih.gov/Blast.cgi).

### Multilocus sequence typing and capsular serotype

Multilocus sequence typing (MLST) assessed in reference to seven housekeeping genes (*gap*A, *inf*B, *mdh*, *pgi*, *pho*E, *rop*B, and *Ton*B). The PCR amplification conditions were based on a protocol obtained from the Institut Pasteur database (www.pasteur.fr/mlst). The *wzi* gene was used for capsular serotyping of KP. The primers used are listed in **Supplementary Table 1**. The PCR- amplified products were sequenced (Sangon Biotech, Shanghai, China), and allelic profiling and determination of sequence types (STs) were confirmed using the Institut Pasteur database.

### Qualitative and quantitative detection of siderophores

The siderophores detection protocol was based on the previous protocol (Han et al, 2022). A single colony on a Columbia blood plate was inoculated onto Modified King B (MKB) solid medium and incubated overnight at 37°C. After iron starvation treatment, the colonies were taken and prepared into a bacterial suspension at a concentration of OD600=1.0 with phosphate-buffered saline (PBS). After adding 10 μL of bacterial suspension to the pre-prepared Chrome Azurol S (CAS) solid medium, the mixture was cultured at 37°C for 2–3 days. After adding 50 μL of bacterial suspension to 5 mL of pre-prepared CAS liquid medium, the mixture was cultured at 37°C for 48h. Following culture, 1 mL of the bacterial solution was removed and centrifuged at 10000 r/min for 10min. Subsequently, 300 μL of supernatant was removed and mixed with CAS detection solution in equal volumes, before detecting the OD630, using CAS culture medium as a control. Quantitative calculation of siderophores was performed as follows: relative siderophore content = [(Ar – As)/Ar] × 100%, where As and Ar represent the OD values of the sample and control groups, respectively.

### Biofilm formation assay

The biofilm formation protocol was modified from the previous protocol (Vuotto et al., 2017) by using 1% crystal violet to dye the biofilm and 95% ethanol to solubilize the dyestuff. The OD was then determined at 570 nm. Each sample was assayed in triplicate and repeated three times. The OD cut-off (ODc) was defined as three standard deviations above the mean OD of the negative control. The results were interpreted as non-biofilm producers (OD≤ODc), weak biofilm (ODc < OD ≤ 2×ODc), moderate biofilm (2×ODc < OD ≤ 4×ODc), or strong biofilm (OD > 4 × ODc) (Vuotto et al., 2017).

### Pulsed-field gel electrophoresis

The PFGE were conducted as previously reported (Du et al, 2017). Bacterial genomic DNA was prepared in agarose plugs and digested with the restriction enzyme XbaI (Promega, USA). The DNA fragments were separated using a CHEF Mappar XA PFGE system (Bio-Rad, USA), with Salmonella enterica serotype Braenderup H9812 used as a comparison standard. The electrophoresis conditions were as follows: 2000–2200 mL 0.5×Tris-boric acid-EDTA buffer, voltage gradient 6 V/cm, electric field angle of 120°, and temperature of 14°C. The electrophoresis parameters were as follows: 5∼15 s, 9.5h, 15∼50 seconds, 9.5h. After electrophoresis, the gel was placed in Gelred dye solution for 25–30 min and stained in pure water for at least 30min, before using a gel imaging system to observe band separation. PFGE patterns were identified using the BioNumerics software (version 5.1, Applied maths, Inc.), and a clustering tree was constructed.

### Plasmid sequencing

The plasmids of CRKP strains were extracted based on the instructions of the plasmid miniprep kit (Omega Bio-tek, Notcross, GA, USA). An Illumina second-generation DNA library was constructed and paired-end sequencing was performed using the Illumina Novaseq6000 (Illumina, USA) high-throughput sequencing platform. The sequencing strategy was Paired-end 150 bp. A PacBio third-generation library was constructed and sequenced using the PacBio Revio system platform and HiFi (High Fidelity) sequencing mode. Plasmid sequence assembly was performed using the Flye assembly software (version: 2.9.6-b1802, https://github.com/mikolmogorov/Flye) (Kolmogorov et al., 2019). Post-assembly polishing was conducted using Pilon v1.24 with Illumina paired-end short-read data to eliminate consensus errors from PacBio HiFi assemblies. Briefly, clean Illumina reads were mapped to draft contigs via Bowtie2 (version: 2.5.4, https://bowtie-bio.sourceforge.net/bowtie2/index.shtml) and processed into sorted indexed BAM with Samtools (version: 1.22.1, http://samtools.sourceforge.net/). Only properly paired mapped reads were retained using samtools view-bF 12 to generate final sorted BAM files. Pilon was run with key parameters ‘--frags final sorted.bam’ to fix single-nucleotide variants and small indels. All other parameters were set to default values. Genome annotation was performed using the NCBI Prokaryotic Genome Annotation Pipeline (https://github.com/ncbi/pgap) (Tatusova et al., 2016). Based on the annotation process, GeneMarkS software (version: 1.14_1.25, http://topaz.gatech.edu/GeneMark/genemarks2.cgi) was used to predict coding genes (Lomsadze et al., 2018). To verify the accuracy of the annotations, the protein and nucleic acid sequences of the annotated genes were compared with the gene sequences of closely related species in the non-redundant protein sequences (nr) and nucleotide database (nt) gene libraries. The plasmid sequences were deposited in GenBank under the accession numbers SRR37213797 (CRKP-6), SRR37213810 (CRKP-38), SRR37213800 (CRKP-39), SRR37213799 (CRKP-41), SRR37213798 (CRKP-48), SRR37213796 (CRKP-68), SRR37213795 (CRKP-95), SRR37213811 (CRKP-101), SRR37213801 (CRKP-144), SRR37213806 (HCKP-4), SRR37213805 (HCKP-9), SRR37213803 (HCKP-13), SRR37213804 (HCKP-14), SRR37213809 (HCKP-67), SRR37213808 (HCKP-71), SRR37213807 (HCKP-87), SRR37213794 (HCKP-106), and SRR37213802 (HCKP-196).

Circular plasmid maps were constructed on the online Proksee server (https://proksee.ca/). The maximum-likelihood phylogenetic tree was visualized in the online Interactive Tree of Life (iTOL) tool (https://itol.embl.de/), and annotation files were imported to plot linear gene arrangement diagrams adjacent to each species terminal branch. Phylogenetic tree was constructed using the core protein-coding genes shared by 18 species. The nucleotide sequences of core protein-coding genes were aligned by MAFFT software (v7.526, http://www.drive5.com/muscle/). Based on the aligned sequences, a maximum-likelihood phylogenetic tree was built with 1000 bootstrap replicates by IQ-TREE (version: 3.0.1, https://github.com/iqtree/iqtree3/) under parameters of ‘-m MFP -B 1000 -alrt 1000’. The jcvi.graphics.synteny module of JCVI toolkit (https://github.com/tanghaibao/jcvi) was used to generate linear collinearity plots of target gene region, with horizontal tracks representing each species’ gene features and gray connecting lines indicating conserved gene pairs. Comparative linear maps of plasmids were generated using EasyFig (version: 2.2.5).

### Mouse infection assay

A mouse infection model was modified from the previously published studies (Han et al, 2022; Xie et al, 2021) to evaluate the virulence discrepancy of representative strains HCKP-67 and CRKP-144 strains. Kunming KM female mice (age, 4∼6 weeks; weight, 18∼22 g) were obtained from the Animal Facility of Kunming Medical University. All mice were housed in a specific pathogen-free (SPF) facility at 24°C temperature and 45∼55% humidity and were always provided with autoclaved food and water. Six mice (n = 6) were randomly allocated into each experimental group and infected with 1.1×10^8^ cfu/mL of each isolate by intraperitoneal injection. Each experimental group was injected with the same concentration of bacterial solution, whereas the blank group was injected with an equal volume of PBS. The survival of mice in each group was recorded for 5 days (120h) after infection. Survival rates were described using Kaplan–Meier curves. Inflammatory cytokines (interleukin [IL]-1β, IL-6, and TNF-α) were detected according to the manufacturer’s instructions for commercial enzyme-linked immunosorbent assay kits (Hangzhou Multi Sciences [Lianke] Biotech Co., Ltd., Hangzhou, China). The lungs were stained with hematoxylin and eosin (H&E) and assessed under a light microscope. All remaining mice were euthanized. The method of euthanasia was cervical dislocation following deep anesthesia. The dose of anesthetics was ketamine 80 mg/kg + xylazine 5 mg/kg.

### Statistical analysis

All statistical analyses were performed using IBM SPSS Statistics for Windows (version 27.0; IBM Corporation, Armonk, NY, USA). Figures were generated using Prism 9 (GraphPad Software Inc., San Diego, CA, USA). Measured data with a normal distribution are expressed as the mean ± standard deviation. The homogeneity of variance of data between the two groups was compared using Student’s t-test. Kaplan–Meier curves were generated for survival analysis, and the log-rank test was used to identify differences in survival between groups. A probability (p) value < 0.05 was considered statistically significant.

## Results

### Clinical data collection

The strains were collected from different departments of the First Affiliated Hospital of Kunming Medical University, including the Geriatric Respiratory Department, Trauma Center, Emergency Intensive Care Unit, Neurosurgery, Transplant Center, and Intensive Care Unit. Among the 18 included strains, 13 were isolated from males and five from females, predominantly from middle-aged and elderly populations. The specimen types included sputum, alveolar lavage fluid, blood, secretion, drainage fluid, and urine. The clinical data are shown in **Supplementary Table 2**.

### Antimicrobial susceptibility test results

Antimicrobial susceptibility tests revealed that 18 strains exhibited high resistance to penicillin, cephalosporins, β-lactamase inhibitors, carbapenems, and quinolones, while remaining sensitive to tigecycline and ceftazidime-avibactam. Nine non-HCKP strains showed different levels of resistance to amikacin, gentamicin, and trimethoprim/sulfamethoxazole, with resistance rates of 44.4%, 44.4%, and 55.6%, respectively, whereas HCKP strains were resistant to these antibacterial drugs. Two colistin-resistant strains (HCKP-67 and HCKP-87) were also identified. All HCKP strains, except for colistin, exhibited a completely consistent antimicrobial resistance phenotype. The antimicrobial susceptibility results are shown in **Supplementary Table 3**.

### Genotypic analysis

**Figure 1:**
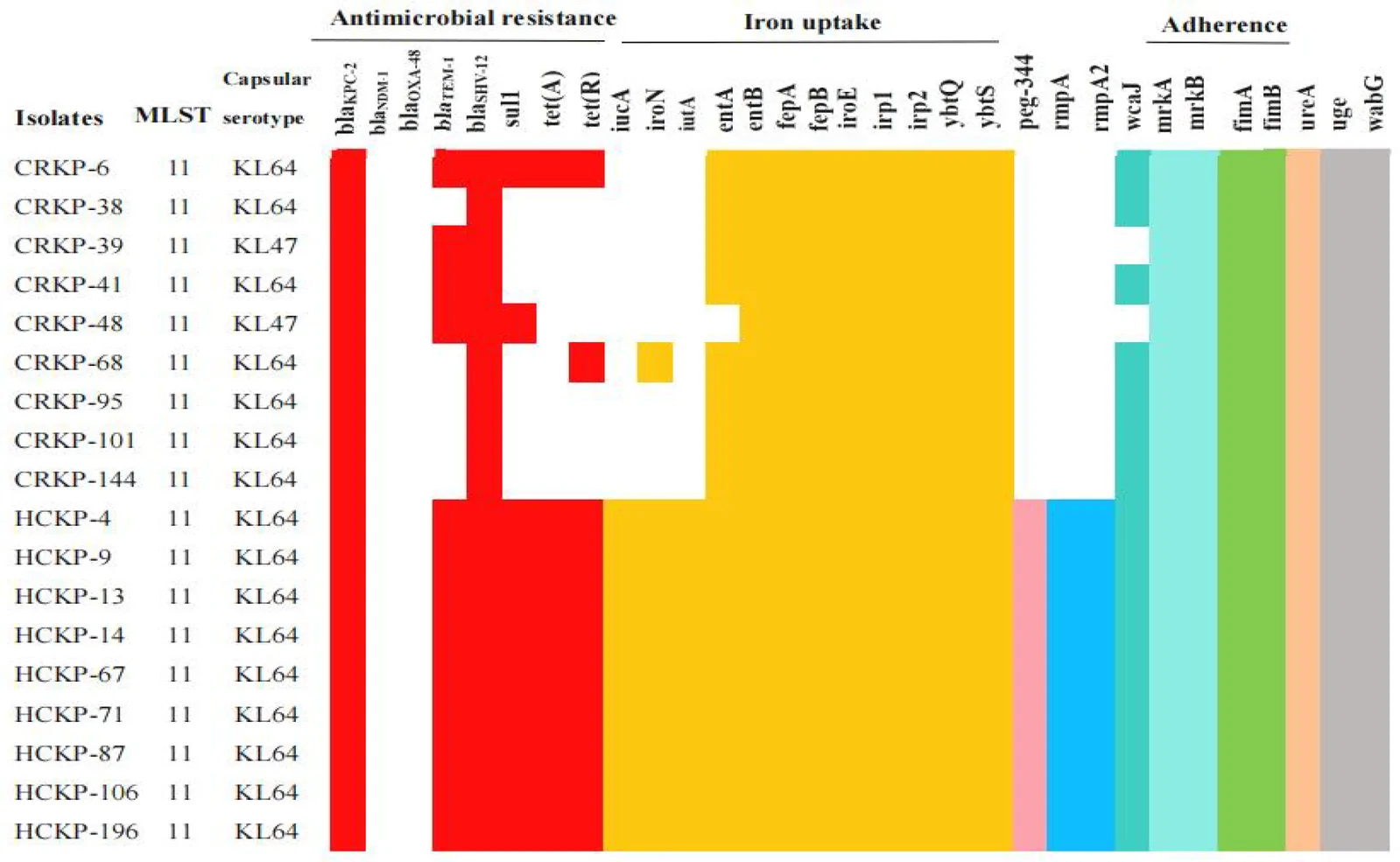
Distribution of antimicrobial resistance genes and virulence genes in non-HCKP and HCKP clinical isolates.

Sixteen genes, including *bla*_KPC-2_, *bla*_SHV-12_, *ent*B, *fep*A, *fep*B, *iro*E, *irp*1, *irp*2, *ybt*Q, *ybt*S, *mrk*A, *mrk*B, *fim*A, *fim*B, *uge* and *wab*G, were identified across the eighteen strains. Whereas thirteen genes, including *bla*_TEM-1_, *sul*1, *tet*(A), *tet*(R), *iuc*A, *iroN*, *iut*A, *ent*A, *peg-*344, *rmp*A, *rmp*A2, *wca*J and *wab*G, were variable, among them, *iuc*A, *iut*A, *peg-*344, *rmp*A, and *rmp*A2 were only detected in HCKP strains, while *iroN* was detected in HCKP strains and also in one non-HCKP strain. The genes *bla*_NDM-1_ and *bla*_OXA-48_ were not detected in all strains. The carbapenem resistance of strains was mediated by the *bla*_KPC-2_ gene. Virulence factors related to iron uptake, adherence, capsule synthesis, regulation, biofilm formation, lipopolysaccharide and urease. The results of gene amplification are shown in **Figure 1**.

### Multilocus sequence typing and capsular serotype

All strains were identified as belonging to ST11 (*gap*A-*inf*B-*mdh*-*pgi*-*pho*E-*rpo*B-*ton*B allele number 3-3-1-1-1-1-4). Based on the wzi allele, the strains were classified into two serotypes namely KL47(allele number 209) and KL64 (allele number 64), among which, the KL64 being the predominant type. The results of MLST and capsular serotype are shown in **Figure 1**.

### Qualitative and quantitative detection of siderophores

**Figure 2:**
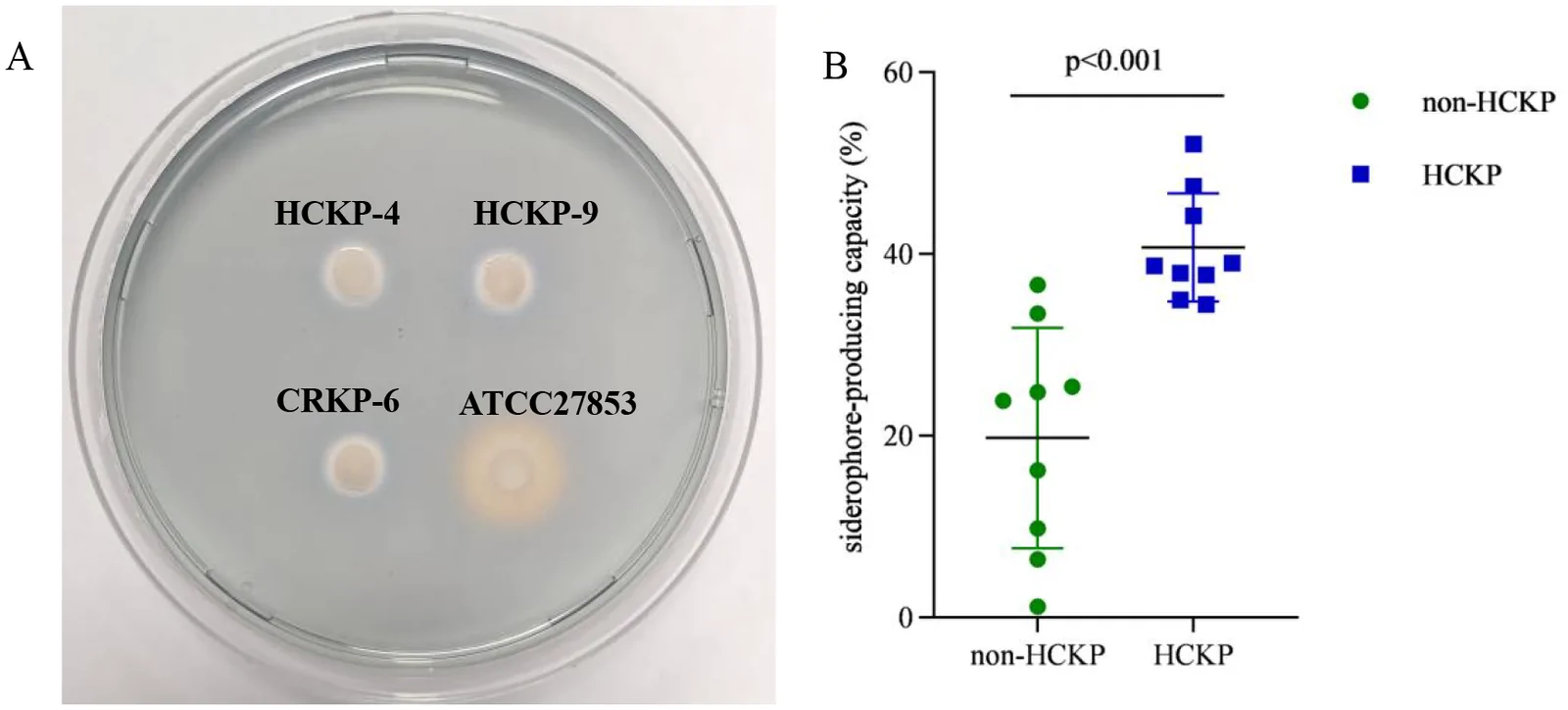
**(A)** Qualitative detection of siderophores of partial strains in CAS solid medium. **(B)** Quantitative detection of siderophores.

Qualitative results of siderophores showed that all strains could produce yellow chelation rings with slightly inconsistent color depth and size, indicating that they all produced siderophores. Compared with the positive control formed by the standard strain *Pseudomonas aeruginosa*ATCC27853, the color of the chelation circles formed by the clinical strains were all lighter, as shown in **Figure 2A**.

The siderophore-producing capacity of the HCKP strain was significantly stronger than that of the non-HCKP strain (p < 0.001), as shown in **Figure 2B**.

### Biofilm-forming capacity

**Figure 3:**
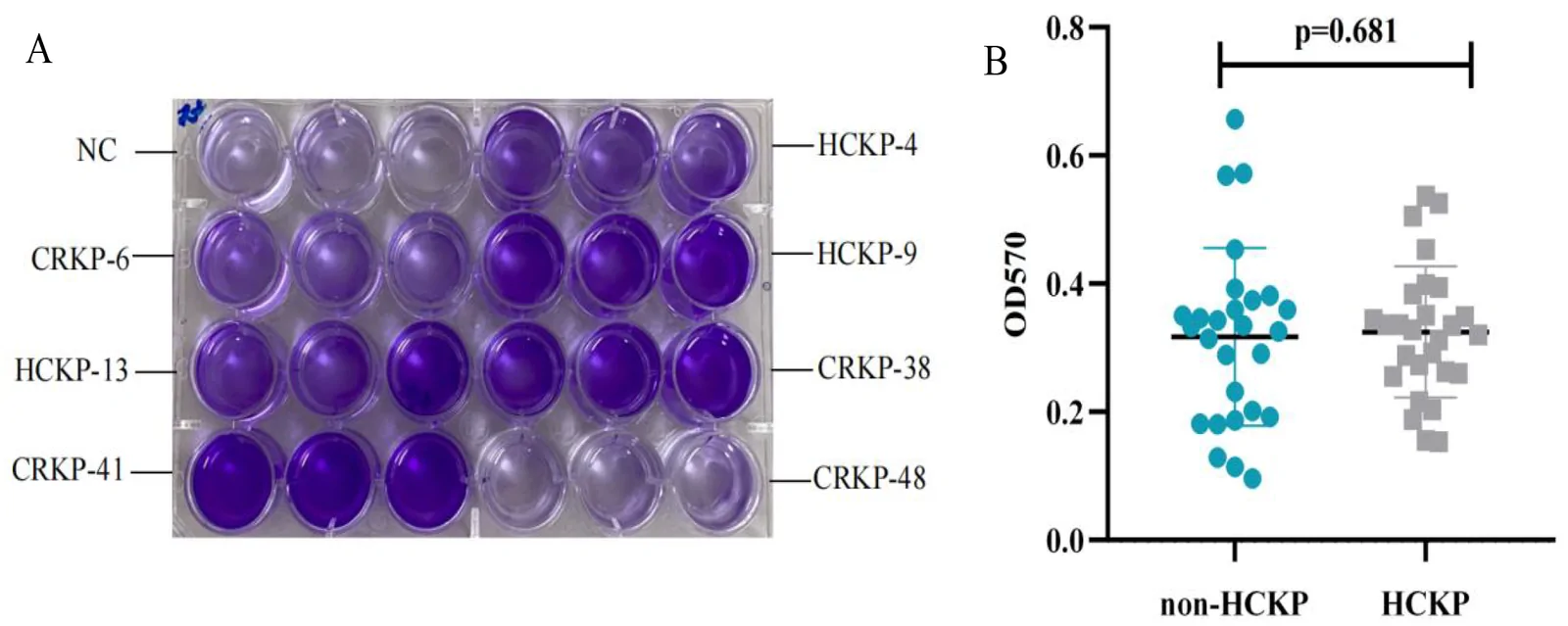
**(A)** Crystal violet staining results of partial strains, with three replicate wells per strain. **(B)** Comparison of biofilm formation ability between non-HCKP and HCKP strains, with three replicate wells per strain. .

The strains in the non-HCKP and HCKP groups exhibited different crystal violet staining results, as shown in **Figure 3A**. The ODc was 0.122, 2×ODc was 0.243, and 4×ODc was 0.487. The mean OD_570_ nm values of the strains CRKP-6∼144 were 0.190, 0.397, 0.355, 0.599, 0.113, 0.202, 0.313, 0.325, and 0.359, while those of HCKP-4∼196 were 0.216, 0.372, 0.523, 0.379, 0.286, 0.322, 0.329, 0.175, and 0.319. According to the interpretation criteria of biofilm formation ability, in the non-HCKP strains, two were weak biofilm producers, six were moderate biofilm producers, and one was a strong biofilm producer. Among the HCKP strains, one showed non-biofilm producers, two were weak biofilm producers, five were moderate biofilm producers, and one was a strong biofilm producer. The two type strains showed no significant difference in biofilm formation ability (*p*=0.681), as shown in **Figure 3B**.

### PFGE analysis

**Figure 4:**
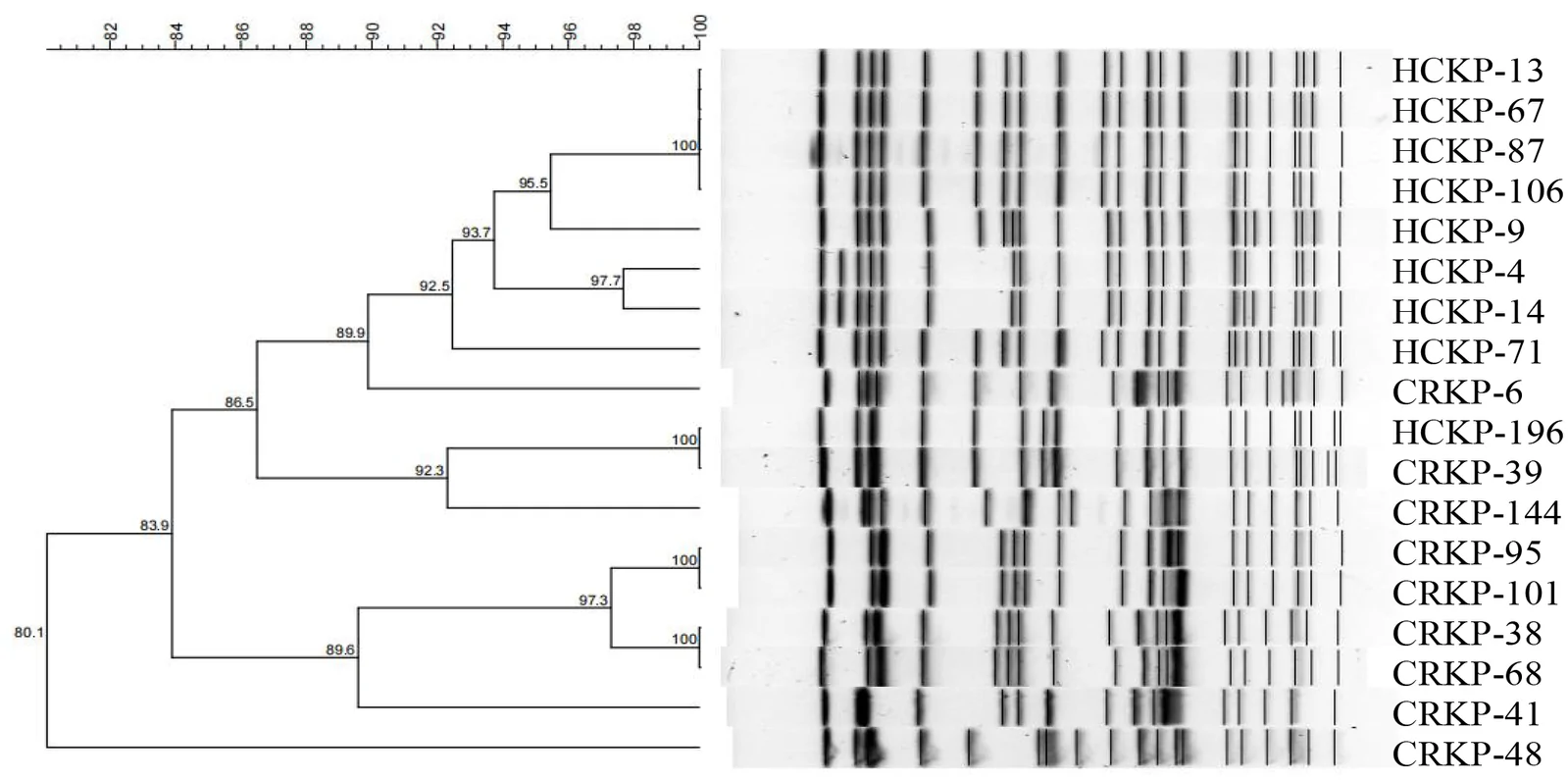
Pulsed-field gel electrophoresis clustering pattern of clinical isolates.

PFGE cluster analysis showed that nine HCKP isolates were divided into five banding types, and non-HCKP were divided into six banding types, respectively, indicating a clonal transmission trend among some strains of both HCKP and non-HCKP, as shown in **Figure 4**.

### Plasmid sequencing analysis

#### Structural analysis of the pLVPK-like plasmid

**Figure 5:**
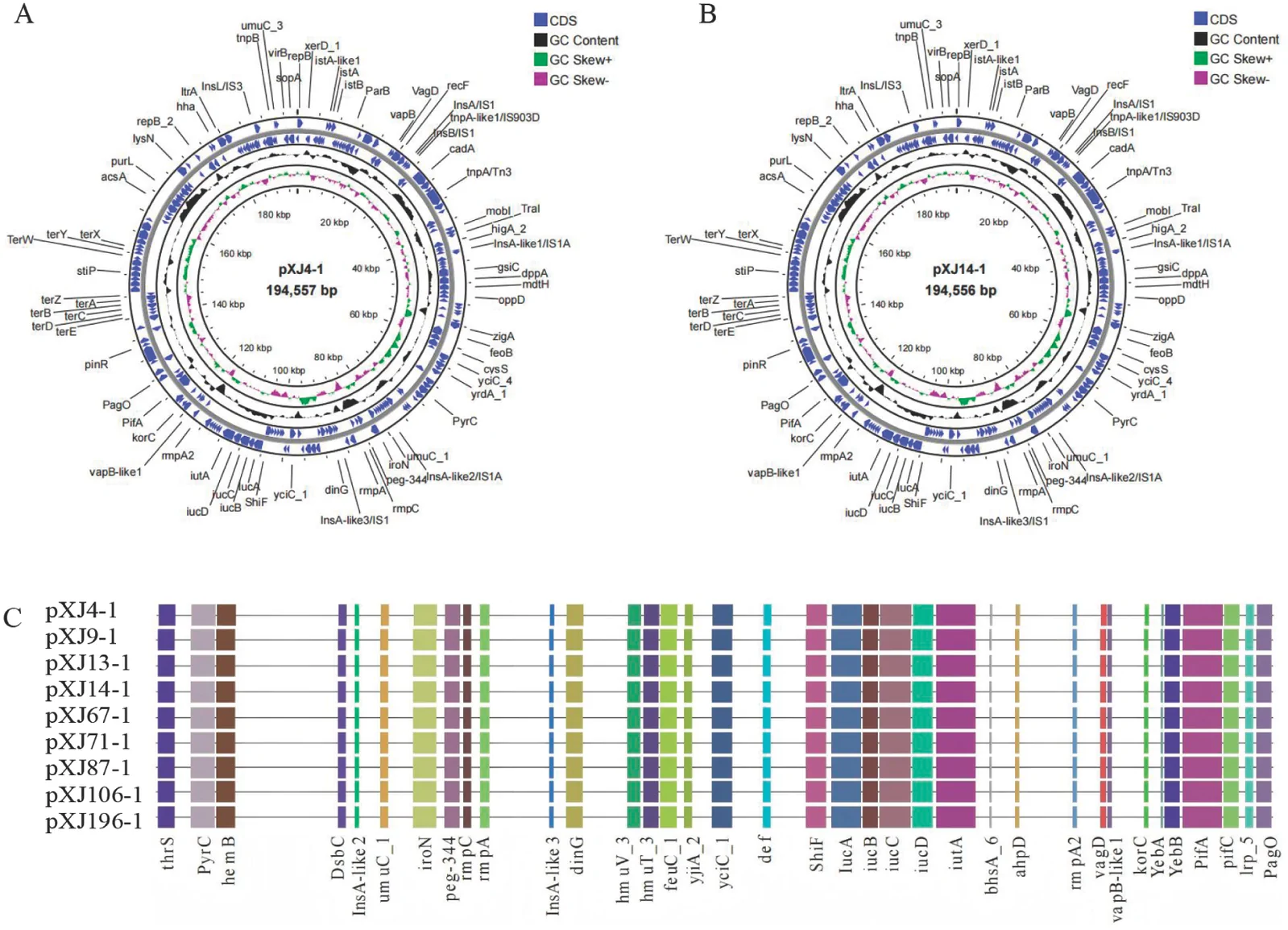
Structural analysis diagram of the virulence plasmid in HCKP strains. **(A, B)** Circular diagrams of the pXJ4-1 (virulence plasmid carried by HCKP-4 strain) and pXJ14-1 (virulence plasmid carried by HCKP-14 strain). From outer to inner, the first circle shows the gene distribution in the virulence plasmid. The second circle represents the GC Content. The third circle represents the GC-skew distribution. **(C)** Linear comparison diagram of the characteristic region of the virulence plasmid of the HCKP strains. Colors indicate different genes.

The HCKP strains carried a pLVPK-like virulence plasmid with a size of approximately 194 to 200 kbp. The structure of this plasmid was highly conserved and harbored the virulence genes *feo*B, *per*R, *iro*N, *peg*-344, *rmp*C, *rmp*A, *iuc*A, *iuc*B, *iuc*C, *iuc*D, *iut*A, *rmp*A2, *Yad*A, *hha*, *ltr*A and *vir*B, as well as the tellurite resistance operon *ter*. Several mobile elements were also identified, including Is*tA-like1*, Is*tA*, Is*tB*, InsA, InsB, tnpA-like1, tnpA, InsA-like1, InsA-like2, InsA-like3, tnpB, and a combination of transfer elements traI. This type of plasmid lacks a complete plasmid conjugation-transfer operon. Notably, they were missing the *iro*BCD genes but retained the *iro*N gene. PCR confirmed the absence of *iro*BCD genes (**Figure 5**).

#### Structural analysis of the bla_KPC-2_-bearing plasmid

**Figure 6:**
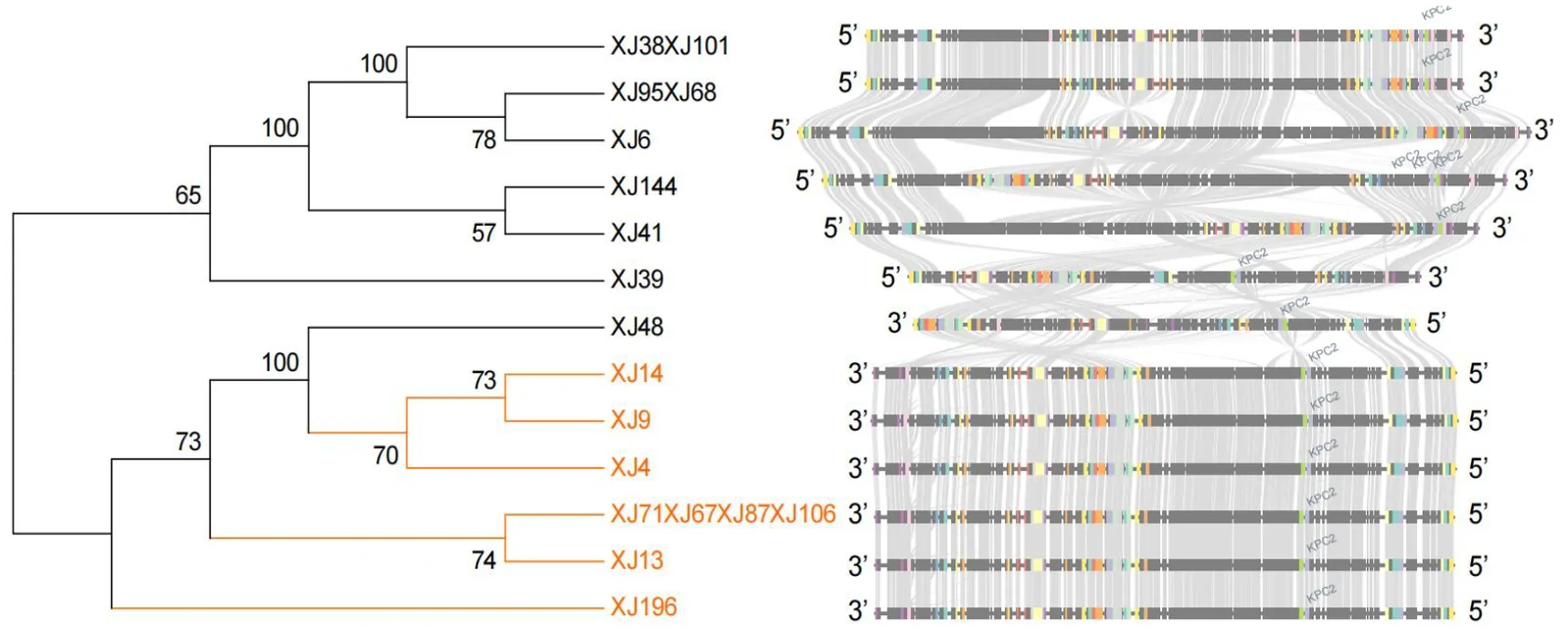
Linear comparison diagram of the characteristic gene regions of the *bla*_KPC-2_-bearing plasmids in HCKP and non-HCKP clinical isolates.XJ38, XJ101, XJ95, XJ68, XJ6, XJ144, XJ41, XJ39, and XJ48 were *bla*_KPC-2_-bearing plasmids in non-HCKP strains, while XJ14, XJ9, XJ4, XJ71, XJ67, XJ87, XJ106, XJ13, and XJ196 were blaKPC-2-bearing plasmids in HCKP strains. Arrowheads represent genes, colors indicate gene function classification. Gray shading represents regions of homology.

**Figure 7:**
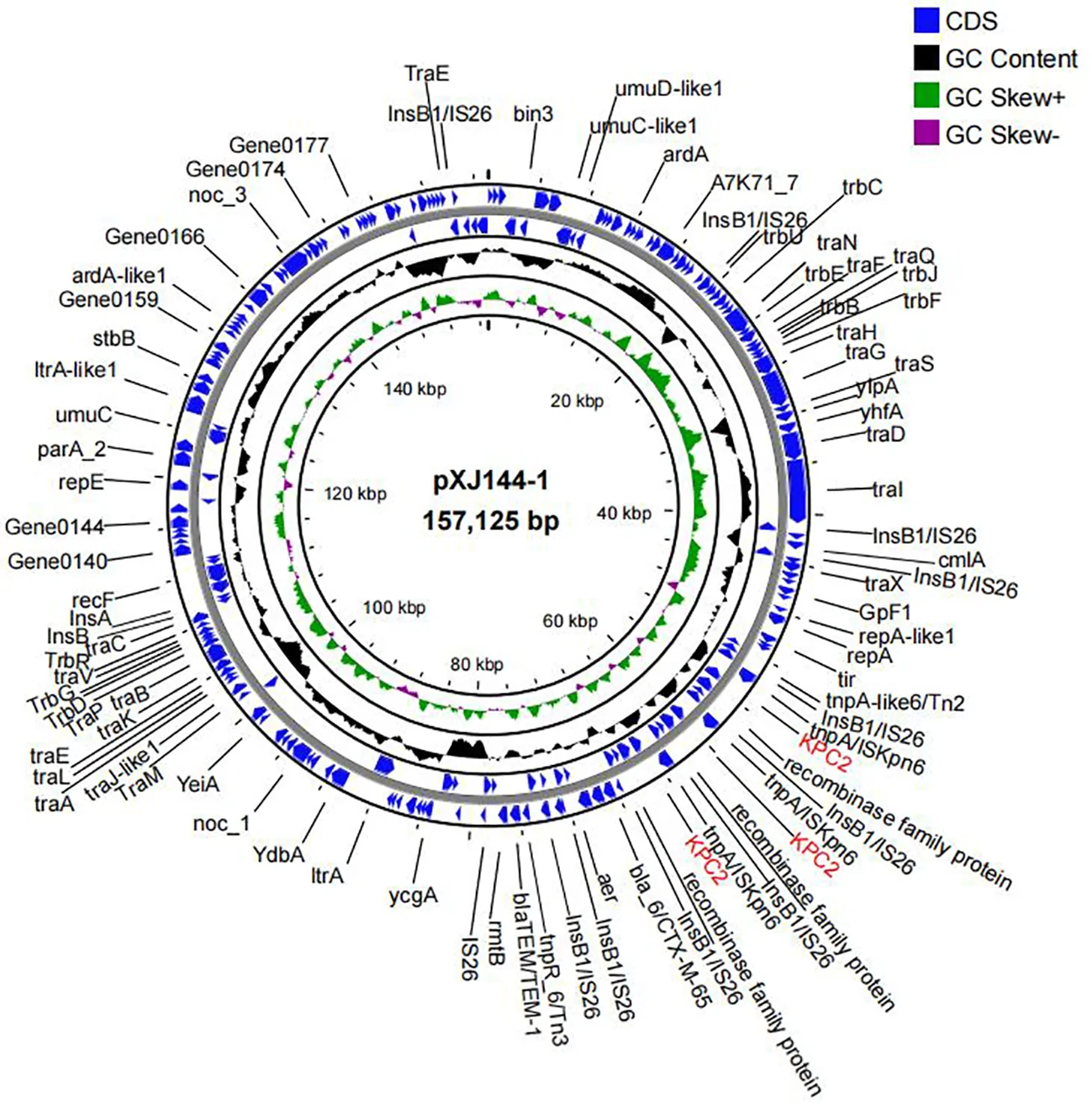
Circular diagrams of the *bla*_KPC-2_-bearing plasmid in the CRKP-144 isolate. From outer to inner, the first circle shows the gene distribution in the plasmid pXJ144-1. The second circle represents the GC Content. The third circle represents the GC-skew distribution.

In HCKP strains, the size of *bla*_KPC-2_-bearing plasmids were ~13kbp, and the G+C content was 53.33%. The mobile elements including Tn3, IS*26*, IS*903B*, TnAs1, IS*Kpn6*, and IS*Kpn27* were identified. The *bla*_KPC-2_-bearing plasmid harbored multiple drug resistance genes, including *bla*_CTX-M-65_, *bla*_TEM-1_, *rmt*B, *bla*_SHV-12_, and *bla*_KPC-2_. Three types of heavy metal resistance gene clusters have been identified: The mercury resistance operon merEDACPTR, the copper resistance operon pcoSRDCBA, and the copper resistance operon silPABCRSE. The conjugation transfer gene clusters traMJLEIX was discovered in all *bla*_KPC-2_-bearing plasmids (HCKP and non-HCKP). Notably, in nine HCKP strains, the structure of these plasmids was highly conserved, with identical mobile elements and resistance genes (**Figure 6**). In addition, the key carbapenem resistance gene *bla*_KPC-2_ was located in the genetic context with insertion sequence IS*Kpn27* upstream and IS*Kpn6* downstream (IS*Kpn6*-*bla*_KPC-2_-IS*Kpn27*). In non-HCKP strains, the structure of the *bla*_KPC-2_-bearing plasmid showed high genetic diversity in the multidrug-resistant region, with resistance factors flanked by multiple insertion sequences, transposons, and integrons, as shown in **Table 1** and **Figure 7**. Compared with HCKP, the “IS*Kpn6*-*bla*_KPC-2_-IS*Kpn27*” structure of non-HCKP was bracketed by IS*26*. Interestingly, in plasmid pXJ-144-1, the three copies of the IS*26*-IS*Kpn6*-*bla*_KPC-2_-IS*Kpn27*-IS*26* unit were present in tandem, as shown in **Figure 7**.

**Table 1:** Genomic features of *bla*_KPC-2_-bearing plasmids in non-HCKP strains.

Isolates	*bla*_KPC-2_-bearing plasmids	Length (bp)	G+C	Mobile genetic elements (MGEs)	Resistance genes
CRKP-6	pXJ6-1	168,249	52.95	Tn2, IS*26*, Tn3, IS*1380*, IS*Kpn27*, IS*Kpn6*, IS*1*, IS*903D*, IS*5075*, Tn6338, intI1, IS*CR1*,IS*Kpn26*	*cml*A, *rmt*B, *bla*_TEM-1_, *bla*_CTX-M-65_, *bla*_KPC-2_, *dhf*rI, *aad*A16, *emr*E, *sul*1, *qnr*B6, *emr*E
CRKP-38	pXJ38-1	137,037	53.08	Tn3, IS*26*, IS*Kpn26*, IS*903D*, IS*1*, IS*Kpn6*, IS*Kpn27*, IS*1380*, IS*903B*	*bla*_KPC-2_, *bla*_CTX-M-65_
CRKP-39	pXJ39-1	117,504	54.63	Tn3, IS*26*, IS*903B*, IS*1*, IS*903D*, IS*Kpn27*, IS*Kpn6*, TnAs1, IS*Kpn26*	*bla*_CTX-M-65_, *fos*A3, *bla*_TEM-1_, *rmt*B, *bla*_SHV-12,_ *mer*EDACPTR, *bla*_KPC-2_, *cml*A
CRKP-41	pXJ41-1	144,287	53.12	IS*26*, IS*1*, IS*903D*, Tn3, IS*Kpn6*, IS*Kpn27*	*bla*_KPC-2_, *bla*_TEM-1_, *rmt*B
CRKP-48	pXJ48-1	61,804	54.12	IS*26*, IS*1*, IS*903D*, Tn7, IS6, IS*Kpn27*, IS*Kpn6*, Tn3	*bla*_KPC-2_, *fos*A3, *bla*_TEM-1_, *rmt*B
CRKP-68	pXJ68-1	137,033	53.07	Tn3, IS*26*, IS*Kpn26*, IS*903D*, IS*1*, IS*Kpn6*, IS*Kpn27*, IS*1380*, IS*903B*	*bla*_KPC-2_, *bla*_CTX-M-65_
CRKP-95	pXJ95-1	137,033	53.07	Tn3, IS*26*, IS*Kpn26*, IS*903D*, IS*1*, IS*Kpn6*, IS*Kpn27*, IS*1380*, IS*903B*	*bla*_KPC-2_, *bla*_CTX-M-65_
CRKP-101	pXJ101-1	137,037	53.08	Tn3, IS*26*, IS*Kpn26*, IS*903D*, IS*1*, IS*Kpn6*, IS*Kpn27*, IS*1380*, IS*903B*	*bla*_KPC-2_, *bla*_CTX-M-65_
CRKP-144	pXJ144-1	157,125	53.02	IS*26*, Tn2, IS*Kpn6*, IS*Kpn27*, IS*903B*, Tn3	*cml*A, *bla*_KPC-2_, *bla*_CTX-M-65_, *bla*_TEM-1_, *rmt*B

#### Structural analysis of the third conservative plasmid type

**Figure 8:**
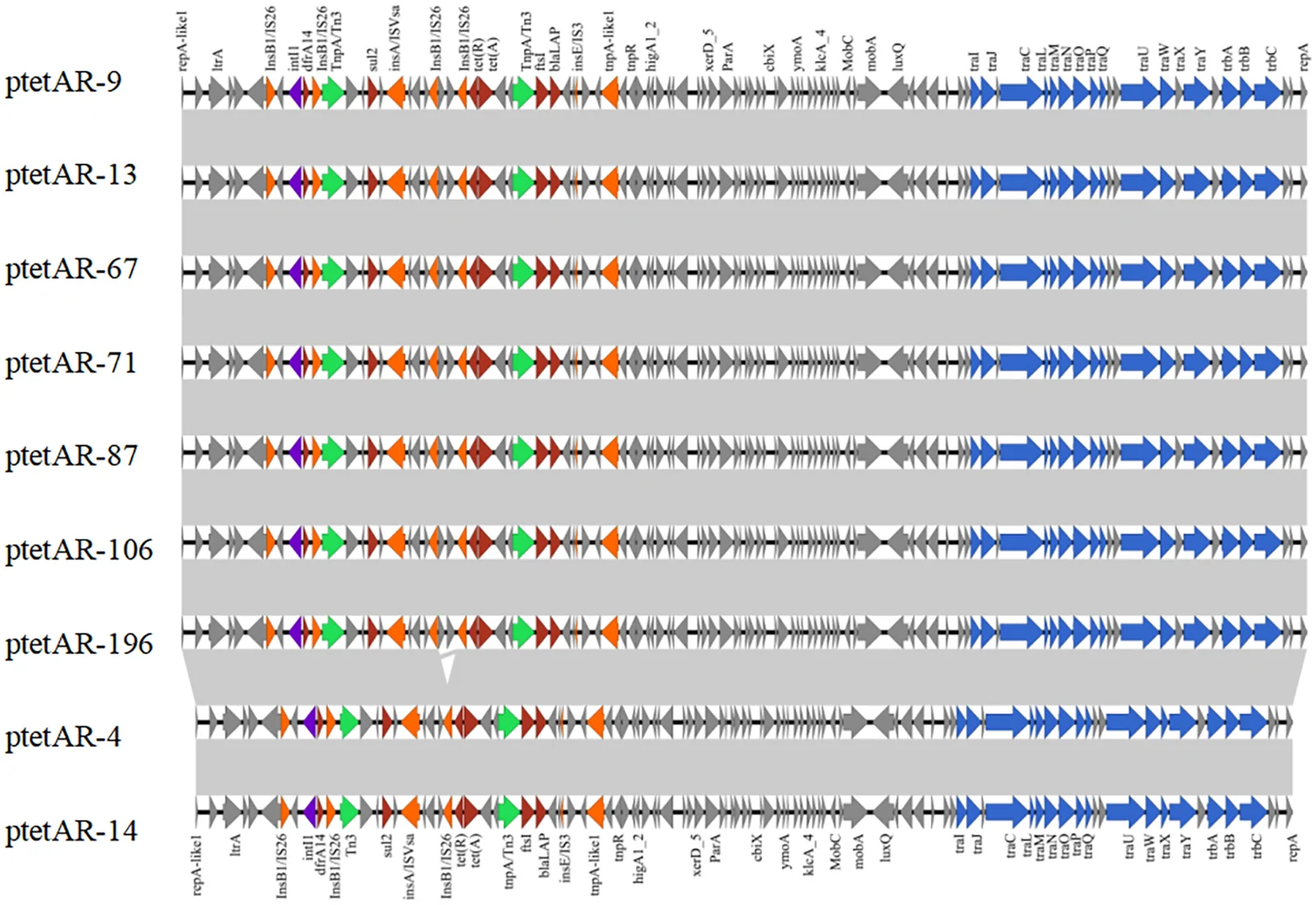
Linear comparison diagram of the characteristic gene regions of the conserved ptetAR plasmid in HCKP strains. Arrowheads indicate genes, with colors representing functional categories and gray shading indicating regions of homology.

A third type of structurally conserved plasmid, designated as the ptetAR plasmid, exists in the HCKP strains due to its carriage of the *tet*(A) and *tet*(R) genes. The plasmid was a resistance plasmid harboring the resistance genes *dfr*A14, *sul*2, *tet*(R), *tet*(A), *fts*I, and *bla*_LAP_, along with mobile elements IS*26*, intI1, Tn3, insA, IS*3*, IS*Kpn19*, and the conjugation transfer gene cluster tra (**Figure 8**). Such plasmids were absent in non-HCKP strains.

### Differences in pathogenicity between HCKP-67 and CRKP-144

#### Survival curves of infected mice

**Figure 9:**
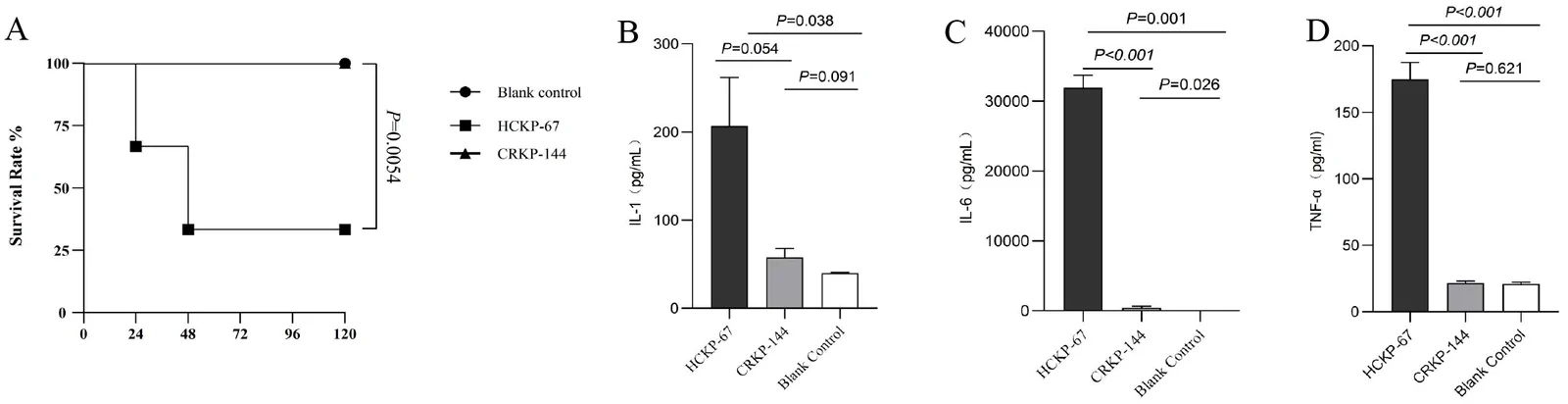
**(A)** Survival curves of mice after injection of bacterial solutions or PBS (blank control group). Serum levels of **(B)** IL-1, **(C)** IL-6, and **(D)** TNF-α in each group.

We constructed a mouse intraperitoneal infection model to evaluate the differences in virulence between HCKP-67 and CRKP-144. Within 5 days, the survival rates of mice in the HCKP-67, CRKP-144, and blank control groups were 33.3%, 100%, and 100%, respectively, with significant differences in survival rates between the HCKP-67 and CRKP-144 groups (p = 0.0054). **Figure 9A** shows the mouse survival curve.

#### Detection of inflammatory factors

As shown in **Figures 9B–D**, among all the investigated inflammatory factors, IL-6 expression was significantly increased in all three groups (HCKP-67, CRKP-144, and blank control). The expression levels of IL-6 and TNF-α were significantly increased in the HCKP-67 group compared with the CRKP-144 group (both p < 0.001) and in the HCKP-67 group compared with the blank control group (p = 0.001, p < 0.001). There was no significant difference in IL-1 expression between the HCKP-67 and CRKP-144 groups.

#### Histopathological observations of mice

**Figure 10:**
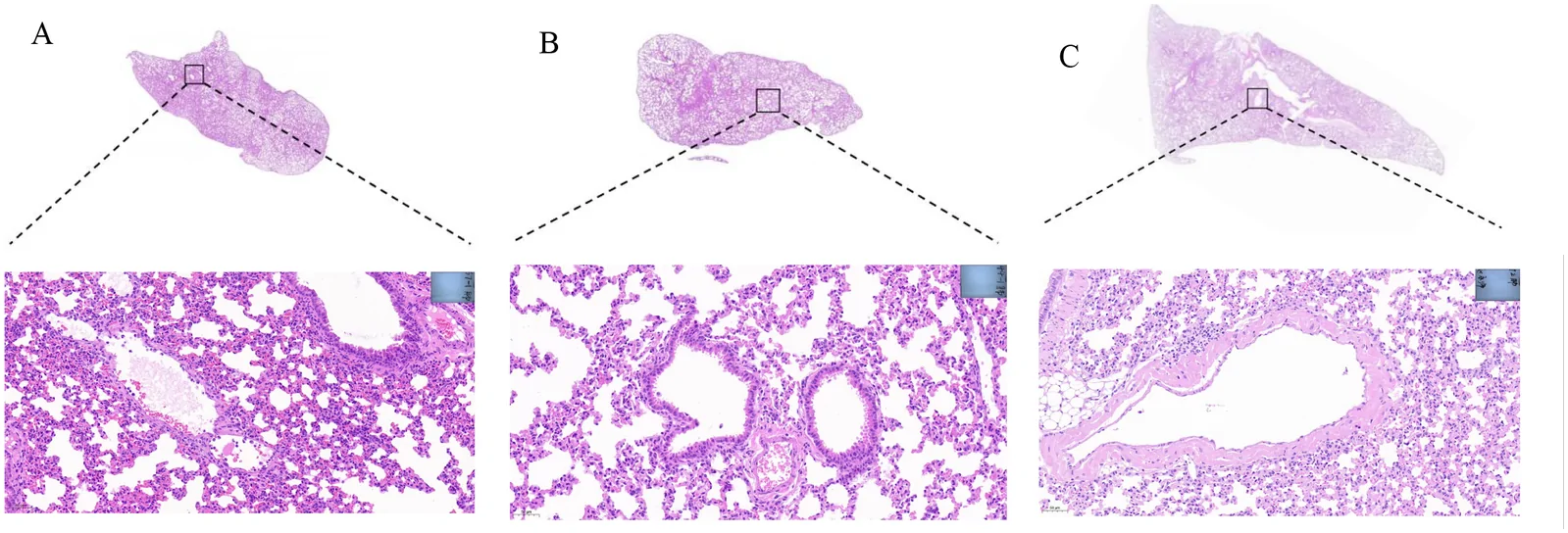
**(A–C)** H&E staining of the lungs of the HCKP-67, CRKP-144, and blank control groups, respectively.

In the HCKP-67 group, changes to the lung tissues of mice included smaller alveolar spaces, wider alveolar intervals, enlarged alveolar epithelial cell nuclei, obvious bleeding, and plasma cell infiltration. In the CRKP-144 group, mouse lung tissue showed mild changes, including essentially normal alveolar lumen size, slightly enlarged nuclei of alveolar epithelial cell nuclei, mild hemorrhage, and occasional plasma cell infiltration. In the blank control group, the alveolar walls of the lung tissue were intact without thickening or inflammatory infiltration, while the interstitium exhibited no edema or congestion. The results of H&E staining of mouse lung tissue are presented in **Figure 10**.

## Discussion

In recent years, various clinically important *Klebsiella pneumoniae* strains, including hvKP, CRKP, and HCKP, have emerged as being responsible for serious infections. HCKP is characterized by the “four highs,” namely high drug resistance, high virulence, high mortality, and high transmissibility (Lei et al., 2024; Zou et al., 2023; Song et al., 2024). The mortality rate of bloodstream infections caused by HCKP has been reported to be as high as 66.7% (Du et al., 2021). Plasmids carrying hypervirulence and carbapenem resistance genes play an important role in HCKP evolution (Xie et al., 2024). To understand the difference between HCKP and non-HCKP, we investigated and compared the microbiological characteristics and plasmid structure of CRKP isolates. Our findings indicate that HCKP has stronger pathogenic capability than non-HCKP, and the virulence and resistance plasmids they carry are highly stable in structure. Furthermore, we discovered a novel pLVPK-like plasmid, which lacks the *iro*BCD virulence gene cluster but retains *iro*N.

Since the first reported case of hepatic abscess complicated by suppurative endophthalmitis caused by the hvKP strain in Taiwan, China, in the 1980s, hvKP has been regarded as the predominant cause of pyogenic liver abscess (Wang et al., 1998; Rossi et al., 2018). However, in this study, the spectrum of infections caused by the evolved HCKP is no longer confined to liver abscesses, it can also lead to infectious diseases such as pneumonia, brain abscess, and cholangitis (**Supplementary Table 2**), which is consistent with the conclusions of previous reports (Wei et al., 2022).

The drug susceptibility test results showed that non-HCKP strains exhibited high-level resistance to penicillins, cephalosporins, carbapenems, and β-lactam/β-lactamase inhibitor combinations (**Supplementary Table 3**). However, some strains (CRKP-38, CRKP-68, CRKP-95, CRKP-101) were sensitive to amikacin and gentamicin. The plasmid sequencing results showed that in non-HCKP strains, the *bla*_KPC-2_-carrying plasmids pXJ38-1, pXJ68-1, pXJ95-1, and pXJ101–1 all lacked the *rmt*B gene. This gene encodes a 16S rRNA methylase, which mediates resistance to aminoglycoside antibiotics such as amikacin and gentamicin (Ma et al., 2009). The drug resistance phenotypes of the remaining HCKP strains were consistent, except for HCKP-67 and HCKP-87, which showed resistance to polymyxin. Unfortunately, the genes responsible for polymyxin resistance were not identified in the plasmids of these two strains. Mutations in the chromosomal genes *mgr*B and *pmr*B can mediate polymyxin resistance (Yap et al., 2022; Lin et al., 2024). Ceftazidime/avibactam is one of the most effective drugs for the treatment of infections caused by *bla*_KPC-2_-producing *Klebsiella pneumoniae*. No drug-resistant strains were found among the strains investigated in this hospital.

In this study, we investigated common resistance and virulence genes in CRKP strains (**Figure 1**). The results showed that the carbapenem resistance phenotype of the strain was mediated by the *bla*_KPC-2_ gene, while other carbapenem resistance genes were not detected. The chromosomally encoded virulence genes *ent*B, *fep*A, *fep*B, *iro*E, *irp*1, *irp*2, *ybt*Q, *ybt*S, *mrk*A, *mrk*B, *fim*A, *fim*B, *uge* and *wab*G had a detection rate of 100% in all isolates, whereas the plasmid-encoded virulence genes *iuc*A, *iut*A, *peg-*344, *rmp*A, and *rmpA2* were only detected in HCKP strains. This difference was mainly mediated by virulence plasmids of HCKP.

Based on the relationship between the capsule locus typing and genetic evolution, ST11-CRKP and its derived HCKP can be divided into two major sublineages: KL47 and KL64. Before 2016, the dominant clinical sublineage of CRKP and HCKP in China was ST11-KL47, which was gradually replaced by the emerging ST11-KL64 HCKP (Zhou et al., 2020; Wu et al., 2024). In this study, only two strains were ST11-KL47 and 16 were ST11-KL64 (**Figure 1**). The predominant type was consistent with the above report.

A biofilm is a protective aggregate formed by bacteria adhering to the surface of objects and enveloped by an extracellular matrix composed of self-secreted polysaccharides and proteins (Sun et al., 2023). Various CRKP strains exhibit a strong capacity to form biofilms, which play crucial roles in both antimicrobial resistance and virulence (Di Domenico et al., 2020; Xie et al., 2024; Li et al., 2024). Siderophores are another important virulence factor of CRKP, which mainly include four major categories, namely, enterobactins, yersiniins, aerobactins, and salmonellins (Arnold, 2024). In this study, we examined the biofilm formation and siderophore-producing abilities of HCKP and non-HCKP (**Figures 2**, **3**). The results showed no significant difference in biofilm formation ability between the two strains, but the siderophore production ability of HCKP was significantly higher than that of non-HCKP. The genes *mrk*A and *mrk*B, both of which are involved in biofilm formation, were expressed at 100% in both HCKP and non-HCKP, consistent with the results of phenotypic tests (Wilksch et al., 2011). The enhanced iron-producing HCKP ability is closely related to the presence of the virulence plasmid (Russo et al., 2024).

The analysis of plasmid structures in HCKP and non-HCKP strains revealed that the high virulence and drug resistance level of HCKP are mainly mediated by the pLVPK-like and *bla*_KPC-2_-carrying plasmids. The structures of pLVPK-like, *bla*_KPC-2_-carrying plasmids, and another associated drug-resistant plasmid in HCKP strains are highly conserved, indicating that HCKP is stable in the hospital environment (**Figures 5**, **6**, **8**). Comparative genomic analysis of the virulence plasmid structure in HCKP strains revealed that the pLVPK-like plasmid in this study lacks the *iro*BCD compared to the previously reported classical pLVPK plasmid (**Figure 5**) (Russo et al., 2024). To validate this result, we designed specific primers to amplify the *iro*BCD, and the amplification results were consistent with the plasmid sequencing results Xinmiao Jia et al. (Jia et al., 2024). found that the loss of *iro*BCDN increased the survival ability of hv-CRKP without decreasing its virulence, thereby endowing it with an evolutionary advantage. However, the virulence plasmid in this study does not carry the *iro*BCD gene but retains the iroN gene. How this affects the evolution, fitness cost, and virulence of HCKP requires further investigation. Multiple mobile elements were identified in the virulence plasmid, indicating that this type of plasmid may evolve into fusion plasmids carrying *rmp*A-*rmp*A2-*peg*344-*iuc*A and antibiotic resistance genes. The pLVPK-like plasmid carries only one conjugative transfer gene (*tra*I), which may be the primary reason for the low frequency of conjugative transfer events involving this plasmid (Xu et al., 2021). In contrast, the presence of multiple insertion sequences (Is*tA-like1*, Is*tA*, Is*tB*) and transposons (tnpA-like1, tnpA, tnpB) provides a foundation for the recombination of the pLVPK-like plasmid structure or the integration of resistance genes.

The *bla*_KPC-2_-carrying plasmid harbors a large number of conjugative transfer genes, which play an important role in the horizontal transfer and trans-species dissemination of this type of plasmid (Cai et al., 2024). The *bla*_KPC-2_-carrying plasmids in non-HCKP strains showed high genetic diversity, especially in the MDR region, with IS*26* insertion elements frequently found adjacent to resistance genes, where they play a critical role in the horizontal transfer of resistance genes and the adaptive evolution of plasmids (**Table 1**; **Figure 6**) (Chen et al., 2025). In plasmid pXJ144-1, three copies of the IS*26*-IS*Kpn6*-*bla*_KPC-2_-IS*Kpn27*-IS*26* unit were present in tandem (**Figure 7**). This unique structure of CRKP is uncommon, and its stability and effects on carbapenem resistance have rarely been reported. HCKP strains harbored a class of highly conserved resistance plasmids (lacking non-HCKP strains). However, whether these plasmids interact with pLVPK-like and *bla*_KPC-2_-carrying plasmids remains to be explored.

Taken together, the low survival rate of mice in the HCKP-67 group, the high expression levels of inflammatory cytokines IL-6 and TNF-α, and the degree of pathological damage in lung tissues all indicated that HCKP-67 had stronger pathogenicity than CRKP-144 (**Figures 9**, **10**). This model supports the correct distinction between HCKP and CRKP and validates the phenotypic and plasmid characteristics described previously.

This study has some limitations. First, the number of strains was small. Second, many interesting findings, such as the role of *iro*BCD deletion with *iro*N retention in the adaptive evolution of HCKP, the impact of the tandem occurrence of the “IS*26*-IS*Kpn6*-*bla*_KPC-2_-IS*Kpn27*-IS*26*” structure on CRKP resistance, and the mechanism underlying the high stability of plasmid structures in HCKP strains, currently remain at the level of phenotypic description and lack in-depth investigation. Third, the sequencing analysis was confined to plasmid sequences without coverage of the whole genome, thus failing to adequately characterize some chromosome-encoded virulence and resistance genes.

In conclusion, HCKP strains are simultaneously of hypervirulence phenotypes and carbapenem resistance and carry resistance and virulence plasmids with conserved core structures, posing a serious threat to public health. Worldwide surveillance of these HCKP strains harboring virulence plasmids with a specific structure (deletion of *iro*BCD and retention of *iro*N) and implementation of stricter control measures are needed to prevent the spread of these strains in hospital settings and evolution to novel highly virulent strains.

## Data Availability

The datasets presented in this study can be found in online repositories. The names of the repository/repositories and accession number(s) can be found in the article/**Supplementary Material**.
