# Supplementary material for: Phenotypic characteristics and plasmid structure differences between ST11 carbapenem-resistant Klebsiella pneumoniae and derived hypervirulent strains
Source: Front Cell Infect Microbiol. 2026 Aug 26;16:1904182. doi: 10.3389/fcimb.2026.1904182 (PMC13553267; doi:10.3389/fcimb.2026.1904182)
Supplement: Supplementary file 1 [file Table1.docx]

**Table 1**. Primers used in the present study

| **Primer name** | **Sequence**（5’～3’） |
| --- | --- |
| *bla*_KPC2_-F | GCTACACCTAGCTCCACCTTC |
| *bla*_KPC2_-R | ACAGTGGTTGGTAATCCATGC |
| *bla*_NDM-1_-F | GGGCAGTCGCTTCCA ACGGT |
| *bla*_NDM-1_-R | GTAGTGCTCAGTGTCGGCAT |
| *bla*_OXA-48_-F | GCGTGGTTA AGGATGAACAC |
| *bla*_OXA-48_-R | CATCAAGTTCAACCCAACCG |
| *bla*_TEM-1_-F | CGTGTCGCCCTTATTCCCTT |
| *bla*_TEM-1_-R | TCCGGTTCCCAACGATCAAG |
| *bla*_SHV12_-F | ACGCTTTCCCATGATGAGCA |
| *bla*_SHV12_-R | CTAGCTCCGGTCTTATCGGC |
| *sul*1-F | GGGCAGATGTGATCGACCTC |
| *sul*1-R | GTCAAAGAACGCCGCAATGT |
| *tet(A)-F* | CCTCAATTTCCTGACGGGCT |
| *tet(A)-R* | TAGAAGCCGCATAGATCGCC |
| *tet(R)-F* | GCGCTTTACTGGCACTTCAG |
| *tet(R)-R* | CGGTCCTTCAACGTTCCTGA |
| *wzi*-F | CGAGCGCTTTCTATCTTGGT |
| *wzi*-R | GAGAGCCACTGGTTCCAGAA |
| *gapA*-F | GTTTTCCCAGTCACGACGTTGTATGAAATATGACTCCACTCACGG |
| *gapA*-R | TTGTGAGCGGATAACAATTTCCTTCAGAAGCGGCTTTGATGGCTT |
| *inf*B-F | GTTTTCCCAGTCACGACGTTGTACTCGCTGCTGGACTATATTCG |
| *inf*B-R | TTGTGAGCGGATAACAATTTC CGCTTTCAGCTCAAGAACTTC |
| *mdh*-F | GTTTTCCCAGTCACGACGTTGTACCCAACTCGCTTCAGGTTCAG |
| *mdh*-R | TTGTGAGCGGATAACAATTTCCCGTTTTTCCCCAGCAGCAG |
| *pgi*-F | GTTTTCCCAGTCACGACGTTGTAGAGAAAAACCTGCCTGTACTGCTGGC |
| *pgi*-R | TTGTGAGCGGATAACAATTTCCGCGCCACGCTTTATAGCGGTTAAT |
| *pho*E-F | GTTTTCCCAGTCACGACGTTGTAACCTACCGCAACACCGACTTCTTCGG |
| *pho*E-R | TTGTGAGCGGATAACAATTTCTGATCAGAACTGGTAGGTGAT |
| *rpoB*-F | GTTTTCCCAGTCACGACGTTGTAGGCGAAATGGCWGAGAACCA |
| *rpoB*-R | TTGTGAGCGGATAACAATTTCGAGTCTTCGAAGTTGTAACC |
| *ton*B-F | GTTTTCCCAGTCACGACGTTGTACTTTATACCTCGGTACATCAGGTT |
| *ton*B-R | TTGTGAGCGGATAACAATTTCATTCGCCGGCTGRGCRGAGAG |
| *rmp*A-F | ACTGGGCTACCTCTGCTTCA |
| *rmp*A-R | CTTGCATGAGCCATCTTTCA |
| *rmp*A2-F | CTTTATGTGCAATAAGGATGTT |
| *rmp*A2-R | CCTCCTGGAGAGTAAGCATT |
| *peg*-344-F | CTTGAAACTATCCCTCCAGTC |
| *peg*-344-R | CCAGCGAAAGAATAACCCC |
| *iuc*A-F | CTCTTCCCGCTCGCTATACT |
| *iuc*A-R | GCATTCCACGCTTCACTTCT |
| *iro*N‑F | CCGCAAAGAGACGAACCGCCTT |
| *iro*N‑R | CGGGCAATCCCCGCTTTGACTT |
| *iut*A‑F | AATCACCTGGGGGCTGGATGCT |
| *iut*A-R | CCGCACCTTCCACGCCGTAAAT |
| *iro*B*-*F | GCCGAGCATGAGCATTCTTG |
| *iro*B-R | TTTTTCGCCACTTCGCTTGG |
| *iro*C-F | TTAACGGTCAGGTTGTCGGG |
| *iro*C-R | GATCTCCGCTTCAGTACCGG |
| *iro*D-F | CGCCAAGAATTAGCGTTCGG |
| *iro*D-R | ACATTGAAGGGGAGTGGCTG |
| *ent*A-F | CTGGATTTTCGCGGTCAGACC |
| *ent*A-R | TTACGCCCCCAGCGTCG |
| *ent*B-F | ATGGCAATCCCTAAATTACAGGCAT |
| *ent*B-R | TTATTGCACCTCGCGGGTGA |
| *fep*A-F | GCGATTTTAGCTTCCGCCTG |
| *fep*A-R | CCCAGCGTCAGGTTCTGATT |
| *fep*B-F | GTGAACTTCTTTTCTTTCTGCCGC |
| *fep*B-R | TTAGCCGAACAGGCTGGAGA |
| *iro*E-F | GTCGCTATCGGCTACCAGAC |
| *iro*E-R | ACCTCGAACATCTGACCGTG |
| *irp*1-F | TGAATCGCGGGTGTCTTATGC |
| *irp*1-R | TCCCTCAATAAAGCCCACGCT |
| *irp*2-F | AAGGATTCGCTGTTACCGGAC |
| *irp*2-R | TCGTCGGGCAGCGTTTCTTCT |
| *ybt*Q-F | GCTACACCTCGGTTGTGGAA |
| *ybt*Q-R | TCTGGCAATGGAAATCCGCT |
| *ybt*S-F | GCGCTCGTTTATGTTCCGTC |
| *ybt*S-R | TAATCGCGTTCAGAGCAGCA |
| *mrk*A-F | TGTCTCCGGTAACCCTGACT |
| *mrk*A-R | ACGTAGTAGGTGAAACGCGC |
| *mrk*B-F | CCTGAACAGCAAAACCGTGA |
| *mrk*B-R | TTAACGGCCACTTTCACTGC |
| *fim*A-F | ATGAAAATCAAAACACTGGCAATGATTG |
| *fim*A-R | TTACTCGTACTGCACTTTGAACGT |
| *fim*B-F | ATGACGAATAACGCCGACATAAAAAA |
| *fim*B-R | CTATTGGATCAGCGACGATCG |
| *wca*J-F | CGGAGTTCCTGTCGTTCCTC |
| *wca*J-R | ACCAAATACTCCACCCACGG |
| *ure*A-F | GCTGACTTAAGAGAACGTTATG |
| *ure*A-R | GATCATGGCGCTACCTCA |
| *uge*-F | GATCATCCGGTCTCCCTGTA |
| *uge*-R | TCTTCACGCCTTCCTTCACT |
| *wab*G-F | ACCATCGGCCATTTCATAGA |
| *wab*G-R | CGGACTGGCAGATCCATATC |

**Table 2.** Clinical data of strains

| Name | Gender | Age | Specimen type | Department | Clinical diagnosis |
| --- | --- | --- | --- | --- | --- |
| CRKP-6 | M | 98 | Sputum | Geriatric Respiratory Department | Chronic obstructive pulmonary disease |
| CRKP-38 | M | 53 | Alveolar lavage fluid | Intensive Care Unit | Severe pneumonia |
| CRKP-39 | M | 38 | Sputum | Intensive Care Unit | Fracture |
| CRKP-41 | F | 81 | Sputum | Intensive Care Unit | Closed severe craniocerebral injury |
| CRKP-48 | M | 71 | Urine | Emergency Intensive Care Unit | Pneumonia |
| CRKP-68 | M | 101 | Blood | Intensive Care Unit | Severe pneumonia with respiratory failure |
| CRKP-95 | F | 89 | Sputum | Geriatric Cardiovascular Medicine | Severe pneumonia |
| CRKP-101 | M | 53 | Alveolar lavage fluid | Intensive Care Unit | Cerebral infarction |
| CRKP-144 | M | 53 | Sputum | Geriatric Respiratory Department | Severe pneumonia |
| HCKP-4 | F | 49 | Sputum | Trauma Center | Acquired facial bone defect |
| HCKP-9 | F | 84 | Urine | Emergency Intensive Care Unit | Severe pneumonia |
| HCKP-13 | M | 73 | Secretion | Emergency Intensive Care Unit | Fever |
| HCKP-14 | M | 49 | Secretion | Emergency Intensive Care Unit | Coma |
| HCKP-67 | F | 44 | Sputum | Neurosurgery | Cerebrospinal fluid leak |
| HCKP-71 | M | 70 | Sputum | Intensive Care Unit | Brain abscess |
| HCKP-87 | M | 48 | Alveolar lavage fluid | Emergency Intensive Care Unit | Severe pneumonia |
| HCKP-106 | M | 83 | Sputum | Neurosurgery | Subarachnoid hemorrhage |
| HCKP-196 | M | 43 | Drainage fluid | Transplant Center | Bile duct stones with cholangitis |

**Table3.** Antibiotic resistance characteristics of strains

|  | Isolates | | | | | | | | | | | | | | | | | | | | | | | | | | |
| --- | --- | --- | --- | --- | --- | --- | --- | --- | --- | --- | --- | --- | --- | --- | --- | --- | --- | --- | --- | --- | --- | --- | --- | --- | --- | --- | --- |
|  | non-HCKP | | | | | | | | |  | HCKP | | | | | | | | | | | | | | | | |
| Antimicrobial susceptibility | CRKP-6 | CRKP-38 | CRKP-39 | CRKP-41 | CRKP-48 | CRKP-68 | CRKP-95 | CRKP-101 | CRKP-144 |  | HCKP-4 | | HCKP-9 | | HCKP-13 | | HCKP-14 | | HCKP-67 | | HCKP-71 | | HCKP-87 | | HCKP-106 | HCKP-196 | |
| Vitek 2 system | | | | | | | | | | | | | | | | | | | | | | | | | | | |
| SCF | ≥64 | ≥64 | ≥64 | ≥64 | ≥64 | ≥64 | ≥64 | ≥64 | ≥64 |  | ≥64 | ≥64 | | ≥64 | | ≥64 | | ≥64 | | ≥64 | | ≥64 | | ≥64 | | | ≥64 |
| CAZ | ≥64 | ≥64 | ≥64 | ≥64 | 32 | ≥64 | ≥64 | ≥64 | ≥64 |  | ≥64 | ≥64 | | ≥64 | | ≥64 | | ≥64 | | ≥64 | | ≥64 | | ≥64 | | | ≥64 |
| CXM | ≥64 | ≥64 | ≥64 | ≥64 | ≥64 | ≥64 | ≥64 | ≥64 | ≥64 |  | ≥64 | ≥64 | | ≥64 | | ≥64 | | ≥64 | | ≥64 | | ≥64 | | ≥64 | | | ≥64 |
| TZP | ≥128 | ≥128 | ≥128 | ≥128 | ≥128 | ≥128 | ≥128 | ≥128 | ≥128 |  | ≥128 | ≥128 | | ≥128 | | ≥128 | | ≥128 | | ≥128 | | ≥128 | | ≥128 | | | ≥128 |
| AMC | ≥32 | ≥32 | ≥32 | ≥32 | ≥32 | ≥32 | ≥32 | ≥32 | ≥32 |  | ≥32 | ≥32 | | ≥32 | | ≥32 | | ≥32 | | ≥32 | | ≥32 | | ≥32 | | | ≥32 |
| FEP | ≥32 | ≥32 | ≥32 | ≥32 | ≥32 | ≥32 | ≥32 | ≥32 | ≥32 |  | ≥32 | ≥32 | | ≥32 | | ≥32 | | ≥32 | | ≥32 | | ≥32 | | ≥32 | | | ≥32 |
| CRO | ≥64 | ≥64 | ≥64 | ≥64 | ≥64 | ≥64 | ≥64 | ≥64 | ≥64 |  | ≥64 | ≥64 | | ≥64 | | ≥64 | | ≥64 | | ≥64 | | ≥64 | | ≥64 | | | ≥64 |
| IMP | ≥16 | ≥16 | ≥16 | ≥16 | ≥16 | ≥16 | ≥16 | ≥16 | ≥16 |  | ≥16 | ≥16 | | ≥16 | | ≥16 | | ≥16 | | ≥16 | | ≥16 | | ≥16 | | | ≥16 |
| ETP | ≥8 | ≥8 | ≥8 | ≥8 | ≥8 | ≥8 | ≥8 | ≥8 | ≥8 |  | ≥8 | ≥8 | | ≥8 | | ≥8 | | ≥8 | | ≥8 | | ≥8 | | ≥8 | | | ≥8 |
| AMK | ≥64 | ≤2 | ≥64 | ≥64 | ≥64 | ≤2 | ≤2 | ≤2 | ≥64 |  | ≥64 | ≥64 | | ≥64 | | ≥64 | | ≥64 | | ≥64 | | ≥64 | | ≥64 | | | ≥64 |
| LEV | ≥8 | ≥8 | ≥8 | ≥8 | ≥8 | ≥8 | ≥8 | ≥8 | ≥8 |  | ≥8 | ≥8 | | ≥8 | | ≥8 | | ≥8 | | ≥8 | | ≥8 | | ≥8 | | | ≥8 |
| SXT | ≥320 | ≤20 | ≥320 | ≤20 | ≥320 | ≤20 | ≤20 | ≤20 | ≤20 |  | ≥320 | ≥320 | | ≥320 | | ≥320 | | ≥320 | | ≥320 | | ≥320 | | ≥320 | | | ≥320 |
| FOX | ≥64 | ≥64 | ≥64 | ≥64 | ≥64 | ≥64 | ≥64 | ≥64 | ≥64 |  | ≥64 | ≥64 | | ≥64 | | ≥64 | | ≥64 | | ≥64 | | ≥64 | | ≥64 | | | ≥64 |
| PB | 1 | 1 | 1 | 1 | 1 | 0.5 | 0.25 | 0.5 | 1 |  | 1 | 1 | | 1 | | 1 | | 64 | | 0.5 | | 8 | | 0.5 | | | 1 |
| Disk diffusion method | | | | | | | | | | | | | | | | | | | | | | | | | | | |
| AMP | 6 | 6 | 6 | 6 | 6 | 6 | 6 | 6 | 6 |  | 6 | 6 | | 6 | | 6 | | 6 | | 6 | | 6 | | 6 | | | 6 |
| SAM | 6 | 6 | 6 | 6 | 6 | 6 | 6 | 6 | 6 |  | 6 | 6 | | 6 | | 6 | | 6 | | 6 | | 6 | | 6 | | | 6 |
| ATM | 6 | 6 | 6 | 6 | 6 | 6 | 6 | 6 | 6 |  | 6 | 6 | | 6 | | 6 | | 6 | | 6 | | 6 | | 6 | | | 6 |
| MEM | 6 | 6 | 6 | 6 | 6 | 6 | 6 | 6 | 6 |  | 6 | 6 | | 6 | | 6 | | 6 | | 6 | | 6 | | 6 | | | 6 |
| GEN | 6 | 18 | 6 | 6 | 6 | 19 | 19 | 18 | 6 |  | 6 | 6 | | 6 | | 6 | | 6 | | 6 | | 6 | | 6 | | | 6 |
| CIP | 6 | 6 | 6 | 6 | 6 | 6 | 6 | 6 | 6 |  | 6 | 6 | | 6 | | 6 | | 6 | | 6 | | 6 | | 6 | | | 6 |
| CZO | 6 | 6 | 6 | 6 | 6 | 6 | 6 | 6 | 6 |  | 6 | 6 | | 6 | | 6 | | 6 | | 6 | | 6 | | 6 | | | 6 |
| CAZ/AVI | 21 | 21 | 23 | 22 | 21 | 22 | 22 | 23 | 23 |  | 22 | 21 | | 21 | | 22 | | 22 | | 21 | | 21 | | 21 | | | 22 |

SCF, Cefoperazone/sulbactam; CAZ, Ceftazidime; CXM, Cefuroxime; TZP, Piperacillin/Tazobactam; AMC, Amoxycillin/Clavulanic acid; FEP, Cefepime; CRO, Ceftriaxone; IMP, Imipenem; ETP, Ertapenem; AMK, Amikacin; LEV, Levofloxacin; SXT, Trimethoprim/Sulfamethoxazole; FOX, Cefoxitin; TGC, Tigecycline; PB, Polymyxin B; AMP, Ampicillin; SAM, Ampicillin/Sulbactam; ATM, Aztreonam; MEM, Meropenem; GEN, Gentamicin; CIP,Ciprofloxacin; CZO, Cefazolin; CAZ/AVI, Ceftazidime/Avibactam.
